# Differences between children with Down syndrome and typically developing children in adaptive behaviour, executive functions and visual acuity

**DOI:** 10.1038/s41598-021-85037-4

**Published:** 2021-04-07

**Authors:** Christine de Weger, F. Nienke Boonstra, Jeroen Goossens

**Affiliations:** 1 Donders Institute for Brain, Cognition and Behaviour, Department of Cognitive Neuroscience, Radboud University Medical Centre Nijmegen, P.O. Box 9101, 6500 HB Nijmegen, The Netherlands; 2 Bartiméus, Institute for the Visually Impaired, Van Renesselaan 309, 3703 AJ Zeist, The Netherlands; 3 Royal Dutch Visio, National Foundation for the Visually Impaired and Blind, Huizen, The Netherlands

**Keywords:** Health care, Cognitive control, Paediatric research, Neuroscience, Neuronal development

## Abstract

In children with Down syndrome (DS) development of visual, motor and cognitive functions is atypical. It is unknown whether the visual impairments in children with DS aggravate their lag in cognitive development. Visual impairment and developmental lags in adaptive behaviour and executive functions were assessed in 104 children with DS, 2–16 years, by comparing their adaptive behaviour, executive functions and visual acuity (distant and near) scores against published age-matched norm scores of typically developing children. Associations between these lags were explored. Mean (± SEM) differences to age-matched norms indicated reduced performance in DS: Vineland Screener questionnaire, − 63 ± 3.8 months; task-based Minnesota Executive Function Scale (MEFS), − 46.09 ± 2.07 points; BRIEF-P questionnaire, 25.29 ± 4.66 points; BRIEF parents’ and teachers’ questionnaire, 17.89 ± 3.92 points and 40.10 ± 3.81 points; distant and near visual acuity, 0.51 ± 0.03 LogMAR and 0.63 ± 0.03 LogMAR (near − 0.11 ± 0.04 LogMAR poorer than distant). Adaptive behaviour (Vineland-S) correlated with the severity of visual impairment (r = − 0.396). Children with DS are severely impaired in adaptive behaviour, executive functions and visual acuities (near visual acuity more severely impaired than distant visual acuity). Larger impairment in adaptive behaviour is found in children with larger visual impairment. This supports the idea that visual acuity plays a role in adaptive development.

## Introduction

Approximately 14.6 in 10,000 children are born with Down syndrome (DS), the most common genetic anomaly^1,2^. They have neurological deficits as well as visual impairments. Both of these may challenge the development of functions that rely on executive control. However, it is unknown whether a relation exists between the visual impairments in children with DS and their lag in cognitive development. Possibly, visual impairments aggravate cognitive development. To study this relation, developmental lags in children with DS need to be specified and quantified. Once we know what may be expected with regard to the development of a child with DS, research on evidence-based interventions for this group could be initiated.

In children with DS, motor, cognitive, practical and social skills develop slower and to a lower level compared with typically developing children^3^. Shortly after birth, there is growth and maturation, but it is slow. In the next several months, the development of neuronal morphology of the visual cortex (where visual information is processed), cerebellar and brain stem size, brain weight, skull size, and visual acuity slows down further^4^. Ocular disorders also limit their visual acuity and visual functioning. These disorders include: frequently occurring and severe refractive errors, nystagmus and accommodation lags (inability to accurately change the shape of the eye lens to focus the image of near objects on the retina)^5,6^.

In children with isolated visual impairment, visual acuity limits the acquisition of skills needed to respond appropriately to environmental demands across a range of contexts, so-called adaptive behaviour and executive functions^7–12^. Severe early-onset visual impairment is considered a major neurodevelopmental disorder. It impacts multiple developmental processes, such as vulnerabilities in motor, cognitive, language, social and attentional domains—all aspects of adaptive behaviour^12^. Studies by Sonckson and Dale^7^, Dale and Sonckson^8^ and Tadic et al.^13^ showed cumulative debilitating consequences on cognitive, language and social skills. Even children with mild to moderate visual impairment show reduced adaptive behaviour. They have more difficulties with skills that affect development and learning than well sighted, typically developing children^11^.

Executive functions are neurocognitive skills that serve as the foundation for early learning. These functions include working memory, control over impulsive thoughts and behaviours, ability to think flexibly and break habits that can get in the way of learning^14^. Differences in parent-rated executive functions were found between school-aged children with all degrees of visual impairment and age-matched typically sighted, typically developing children^10^. Children with severe to profound visual impairment had the greatest difficulties. With teacher-ratings, Heyl and Hintermair^9^ found that visually impaired students also performed poorer at school than typically developing children. Compared to children in mainstream schools, visual impaired children at special schools had even more problems in all domains of executive functions. Almost all domains of behavioural problems and the executive function domains correlated. Although their study may have included some children with DS at special schools, the relation between visual acuity and adaptive behaviour or development of executive functions has not yet been studied in a large cohort of children with DS.

In addition to the learning difficulties associated with the typical malformation of central brain structures in DS, visual impairment could also have an impact on the acquisition of skills needed to respond appropriately to environmental demands. Children with DS attend regular schools where they have to find their way between typically developing children, or they attend special schools where children with other demands surround them. Insight in the development of adaptive behaviour and executive functions of children with DS in combination with their limited visual acuity may contribute to better tailored therapeutic care and guidance at school.

The current study, therefore, compares adaptive behaviour, executive functions and visual acuity in children with DS with published norm scores of typically developing children and analyses possible associations between these different abilities. Adaptive behaviour and executive functions in everyday life were assessed using parents’ questionnaires, Vineland-Screener (Vineland-S)^15,16^, BRIEF-P^17–19^ and BRIEF^20–22^ questionnaires commonly used in DS in clinical practice. The assessment of executive functions was complemented with the teachers’ questionnaire of BRIEF and a task-based test, the Minnesota Executive Function Scale (MEFS)^23,24^. Visual acuities were assessed with symbol discrimination on visual acuity charts, LEA symbols^25^ or Kay pictures^26^.

## Methods

The assessments presented here were part of a Randomized Controlled Trial (RCT) on the effects of wearing bifocal eye glasses in children with DS^27^. The project was conducted in accordance with the tenets of the Declaration of Helsinki. It was reviewed and approved by the Dutch Medical Ethics Committee of the Isala Hospitals (NL48288.75.14/METC: 14.0333) and registered in ClinicalTrials.gov (NTC02241356). For the current cross-sectional study, we used the baseline measurements of this RCT, i.e., before children were randomised in one of the two treatment groups (bifocal or unifocal glasses). The data were collected at 15 participating locations in the Netherlands, 14 hospitals and 1 institution for the visually impaired. Locations were geographically spread across the country, serving both rural and urban populations of diverse social economic status.

Normative data were obtained from studies on typically developing children (see Table 1).

**Table 1:** Normative studies used.

Normative studies	Source	n =	Age range (years)
Vineland-S	Sparrow et al., 1993^15,16^	979	0–6
MEFS	Carlson, 2019^37^	32,800	2–17
BRIEF-P	Gioia et al., 2003^17,18^	1747	2–5
BRIEF parents' version	Huizinga et al., 2009^20,21^	3333	5–17
BRIEF teachers' version	Huizinga et al., 2009^20,21^	941	5–11
Visual acuity (total n = 2985)	Salomao et al., 1995^38^	646	0–2.5
	Pan et al., 2010^39^	1722	2.5–6
	Lai et al., 2011^40^	212	3–6
	Huurneman et al., 2012^36^	75	4–8
	Jeon et al., 2010^41^	78	5–11
	Dobson et al., 2009^42^	252	5–12

Published studies on typically developing children from which we extracted normative data. For each test, the table lists the source of the normative data (i.e., the published study), the number of included children and their age range.

### Participants

Written informed consent was obtained from both parents of each child, or from one parent in case of single parenthood. Inclusion criteria included (1) diagnosis of Down syndrome, (2) age range from 2 to 18 years, (3) ability to respond (verbally or non-verbally) to vision tests if they were older than 5 years. Age two, the age at which most children could sit and look downwards to their toys in their hands, was chosen as youngest age for inclusion. A total of 104 children with DS between 2 and 16 years (23 and 205 months) old were included. The children were recruited from participating locations in cooperation with the Dutch DS foundation and many organizations of medical and allied health professionals who are involved in the medical guidance of children with DS. Participants’ characteristics are listed in Table 2. More details about participants and study design of the RCT are given in our previous papers^27,28^.

**Table 2:** Cohort characteristics.

Cohort characteristics	n =	%	Mean	Standard deviation
Number of children with DS	104			
Boys	51	49		
Children attending school	91	88		
Children not using glasses	28	27		
Nystagmus	17	16		
Manifest strabismus	31	30		
Age (months)			105.3	42.7
Accommodation lag (dioptres)			2.21	0.89
Manifest angle of strabismus (prism dioptres)			21.03	12.5
Distant visual acuity (LogMar)			0.43	0.26
Uncrowded near visual acuity (LogMar)			0.56	0.32
Crowded near visual acuity (LogMar)			0.64	0.29

Data are given either as numbers (n =) and percentages (%) or as mean with standard deviation.

### Assessment procedures

Procedures for assessments of visual functions and executive functions were protocoled. The local investigators, orthoptists from the participating locations, were trained to perform unfamiliar orthoptic tests, to administer the MEFS as prescribed by Reflection Sciences, LLC, and to use the digital research data manager ResearchManager^29^.

First, informed consent and the medical history was obtained from the parent(s). Next, a baseline orthoptic assessment was performed, followed by an assessment of executive functions with the MEFS. Children wore their habitual glasses during these assessments. If the child had no glasses prescribed, assessments were performed without glasses. At the end of the first visit, the BRIEF-P or BRIEF (parents’ and teachers’ versions) and Vineland-S questionnaires were handed out.

If a child became uncooperative, testing was stopped according to the Dutch code of conduct relating to expressions of objection by people who are incapable of giving consent, minors or mentally disabled participating in medical research (Code of conduct in the Netherlands 2002, NVK Code of conduct in the Netherlands 2001). Reasons for missing data, as a result of a lack of cooperation or otherwise, were noted.

#### Adaptive behaviour, Vineland-S questionnaire

Parents were asked to fill out the questionnaire Vineland-Screener (Vineland-S^15,16^, either on paper or online. This questionnaire with 72 items covering the four domains of adaptive behaviour—communication, socialization, daily living skills, and motor skills—was used to estimate the adaptive behavioural age of the child. In typically developing children, the adaptive behavioural age (assessed by their adaptive behaviour in the Vineland-S) equals their calendar age. In our study, we refer to this adaptive behavioural age estimated with Vineland-S questionnaire as ‘Vineland adaptive behavioural age’. Control data were obtained from Sparrow et al.^15^ (n = 979, age range 0–6 years).

#### Executive functions

Executive functions were measured in a task-based test (Minnesota Executive Function Scale, MEFS) and complementary information^30^ was obtained with ratings of contextual executive function performance by the children’s parents and teachers (BRIEF-P and BRIEF questionnaires).

##### Task-based test of executive functions, MEFS

The MEFS is a standardized game-like test to measure executive function and learning readiness in children^24^. It captures the gradual development of executive functions across the entire preschool and subsequent elementary school period. The MEFS measures a combination of attention span, the ability to retain information, behavioural management and flexible thinking. It has been tested in more than 32,800 typically developing children, aged 24–215 months, in the United States and is valid and reliable (Intraclass Correlation: 0.94) across a wide range of executive functions^23,24,31^). The MEFS is an engaging computer card-sorting game that is administered one-on-one with a child. In this rule-switch task, examiners asked the participants to match a card to a target^14,32,33^. First, in teaching trials, the examiner directed the child to match the cards on one dimension (colour; e.g., “green ones go here”, or shape: e.g., “lions go here”). After this, the MEFS test includes 7 levels of increasing difficulty, determined by the rules and the images to sort. The 7 levels (corresponding to test scores 10, 20, 30, 40, 50, 60, ≥ 70) are subdivided in decimals to indicate scores of subsets. This test did not presume the verbal ability of the child and the cards are large enough to be distinguished easily by subjects with reduced (near) vision with a visual acuity of 1.0 LogMAR. The test was administered on an iPad Air (iPad Air 2, 16 GB—Screen with and height, 197 × 147 mm; resolution 1536 × 2560 pixels; pixel pitch 0.077 mm^2^). Children with DS tend to love games on tablets, so we typically obtained good cooperation of the children with the MEFS.

##### Rating-based assessment of executive functions, BRIEF and BRIEF-P questionnaires

To obtain informant report, we asked the parents to fill out one of two questionnaires. Depending on the calendar age of the child, this was either the BRIEF-P (Behavioural Rating Inventory of Executive Function for preschool age, designed for the age range 2–5 years)^17–19^ or the BRIEF (Behavioural Rating Inventory of Executive Function, designed for the age range 5 to 17 years)^20–22^. We also asked the parents to have the teacher fill out the teachers’ questionnaire of the BRIEF (age range 5 to 11 years). The BRIEF and BRIEF-P questionnaires are designed to provide an ecologically valid real-world assessment of executive functions.

In our study, we adjusted the age limits of the BRIEF-P and BRIEF questionnaires to better match the administered questionnaire to the adaptive developmental age of children with DS. The BRIEF-P was intended for participants under the age of 8, whereas the BRIEF was intended for participants older than 8 (see Table 3a). The age limit of 8 years was based on the study of van Gameren et al.^34^ and personal communication with M. Huizinga, author of the Dutch BRIEF questionnaire^21,22^. Van Gameren et al.^34^ found a developmental age in children with DS of half their calendar age but with a wide confidence interval.

The BRIEF-P questionnaires collected ratings on 5 executive functions-scales: inhibition, shift (being flexible in switching allocation of attention), emotional control, working memory, and plan/organize. The BRIEF questionnaires, parents’ and teachers’ versions, collected ratings on 8 executive functions-scales: inhibition, shift (being flexible in the allocation of attention), emotional control, initiate, working memory, plan/organize, organizing of materials, and monitor. For each of the 63 (BRIEF-P) or 86 (BRIEF) statements, parents indicated whether the particular behaviour described in the item had Never, Sometimes, or Often been a problem for their child within the last six months. Teachers did so for 75 (BRIEF) statements. For each of these questionnaire types, we only considered the raw aggregated score across domains, the raw Global Executive Composite (rGEC). Higher scores represent greater levels of executive function impairment.

#### Visual functions

Visual acuities—both at distant and at near—were assessed with their habitual glasses or without glasses if the child did not use glasses. We applied non-verbal or verbal methods (matching or naming LEA symbols^25^ or Kay pictures^26^ on visual acuity charts) according to the capacity of the child. Distant visual acuity was typically tested at 5 m with LEA linearly arranged cards or Kay Pictures. Uncrowded and crowded near visual acuity was assessed binocularly at 40 cm with LEA symbols with absolute spacing^35,36^. Near vision was measured without bifocals, as only baseline measurements were included in this study. In case 40 cm was not feasible because the child insisted to keep the card at a closer distance (n = 13), the actual distance (range 10 to 40 cm) was noted and visual acuity scores were corrected accordingly (although a shorter distance gives less accurate near VA estimates)^36^.

### Data analysis

Statistical analysis was performed using the statistical package for the social sciences (SPSS version 23, IBM Inc., Chicago, IL).

Only questionnaires in which the number of filled in items passed the limits listed in the respective manuals^16,18,21^ were included. We analysed the BRIEF-P, BRIEF parents’ version, and BRIEF teachers’ versions separate from each other.

Because of the expected discrepancy between calendar age and the adaptive behavioural age for children with DS, the BRIEF-P and BRIEF questionnaire data were not transformed into age-adjusted scores. Instead, we used the raw scores (raw Global executive composite score, rGEC) for all our analyses. In this way, relations between age and total difficulties could be evaluated. Subdomains of the Vineland-S or BRIEF-P and BRIEF questionnaires were not analysed separately because the study focus was on a general developmental assessment. We also used the raw MEFS score, the Total Score, as opposed to its norm-referenced score.

Continuous data are summarized by mean, standard deviation (SD) and range—nominal data by frequencies and proportions. Student’s *t*-test and Chi-square test were applied to analyse group differences, respectively.

**Figure 1:**
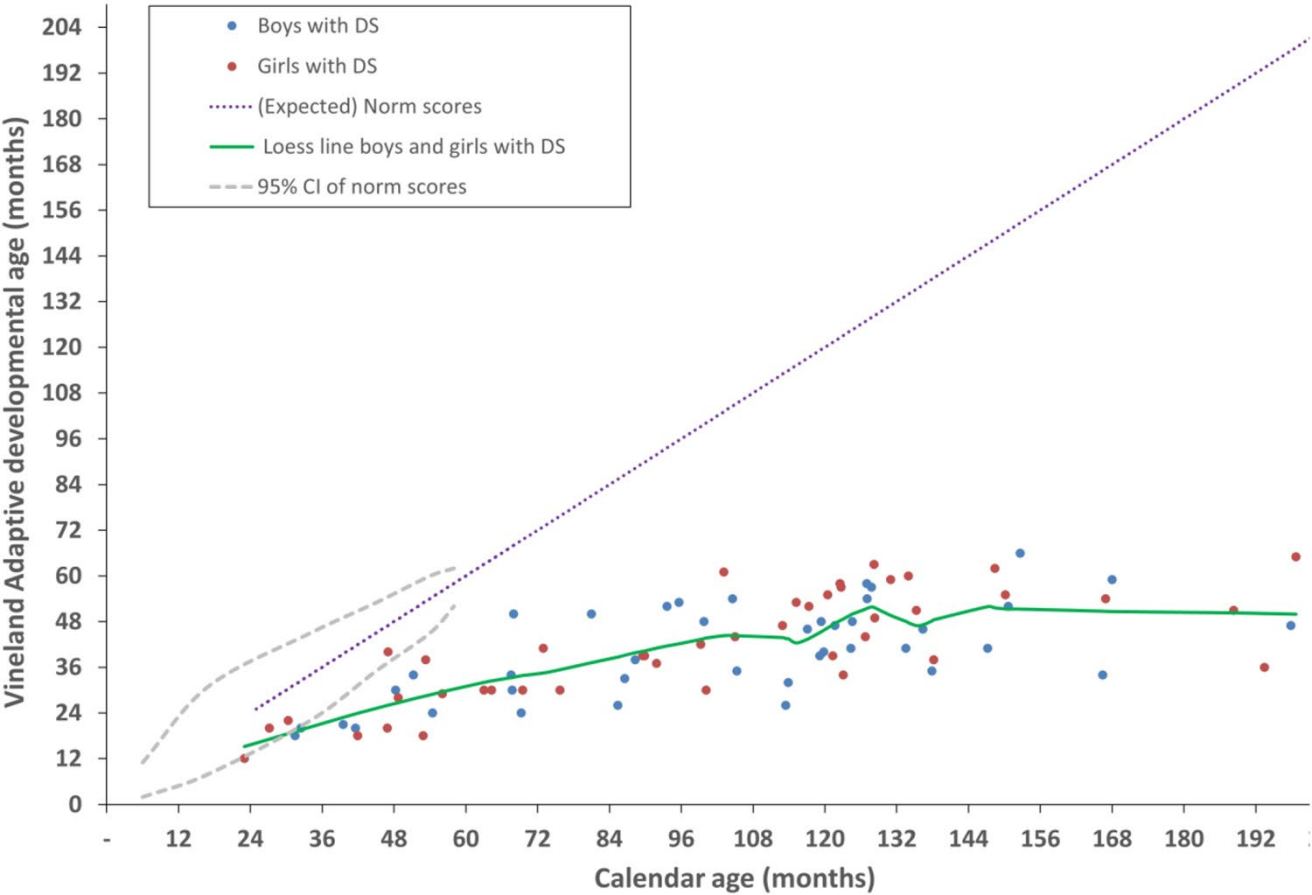
Vineland Adaptive behaviour. Adaptive developmental age as estimated by their adaptive behaviour in the Vineland-S, as a function of calendar age in 83 children with DS. Note that all scores fell below the norm (identity line; n = 979, age range 1–72 months; Sparrow et al.^15,16^). According to the measurement focus of the Vineland-S, the norm scores of typically developing children equal their calendar age. Blue bullets: Measured boys with DS (n = 40). Red bullets: Measured girls with DS (n = 43). Solid green line: Loess line fitted to the data of the children with DS pooled across boys and girls. Dotted purple line: (Expected) norm scores (mean) of typically developing children pooled across boys and girls. Grey dashed lines: upper and lower bound of the 95% confidence interval of norm scores of typically developing children.

**Figure 2:**
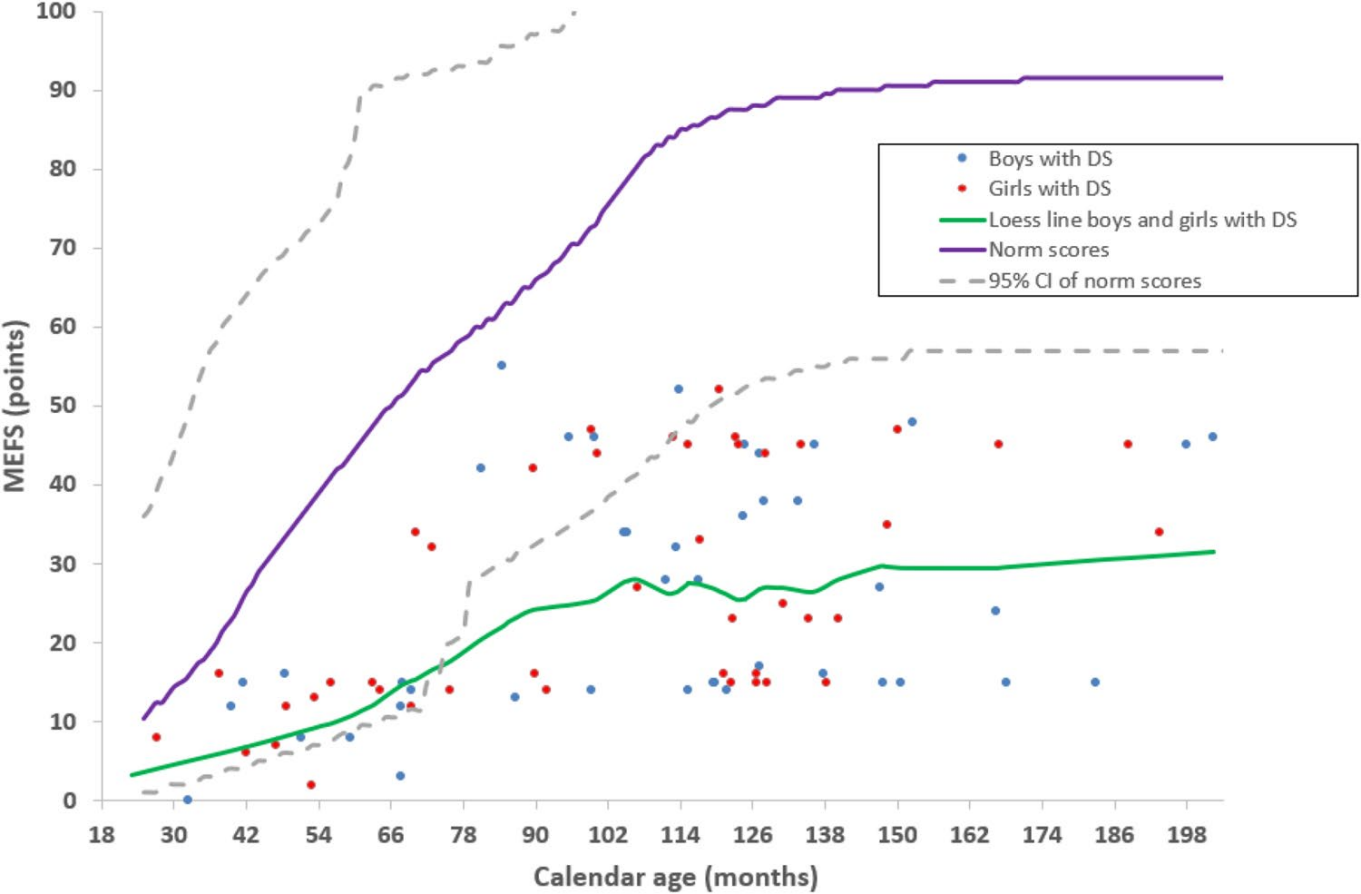
Task-based executive functions. Minnesota Executive Function Scale (MEFS) data of 86 children with DS as a function of calendar age together with normative data (n = 32,800, age range 24–216 months; Carlson^37^). Note lower MEFS scores in children with DS compared to the norm scores. Blue bullets: Measured boys with DS (n = 43). Red bullets: Measured girls with DS (n = 43). Green curve: Loess line fitted to the data of the children with DS pooled across boys and girls. Purple curve: Norm scores (mean) of typically developing children pooled across boys and girls. Grey dashed lines: upper and lower bound of the 95% confidence interval of norm scores of typically developing children.

**Figure 3:**
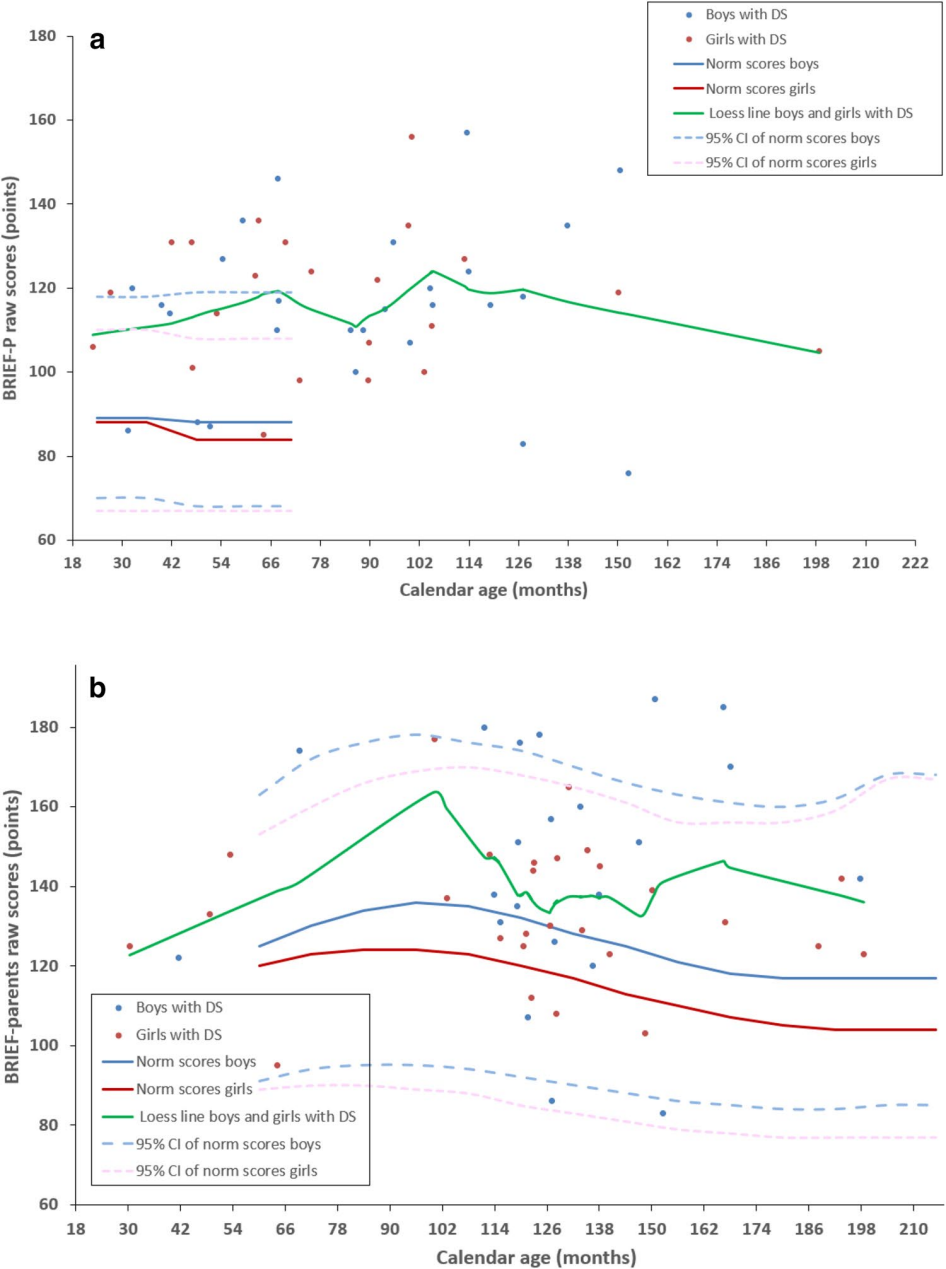

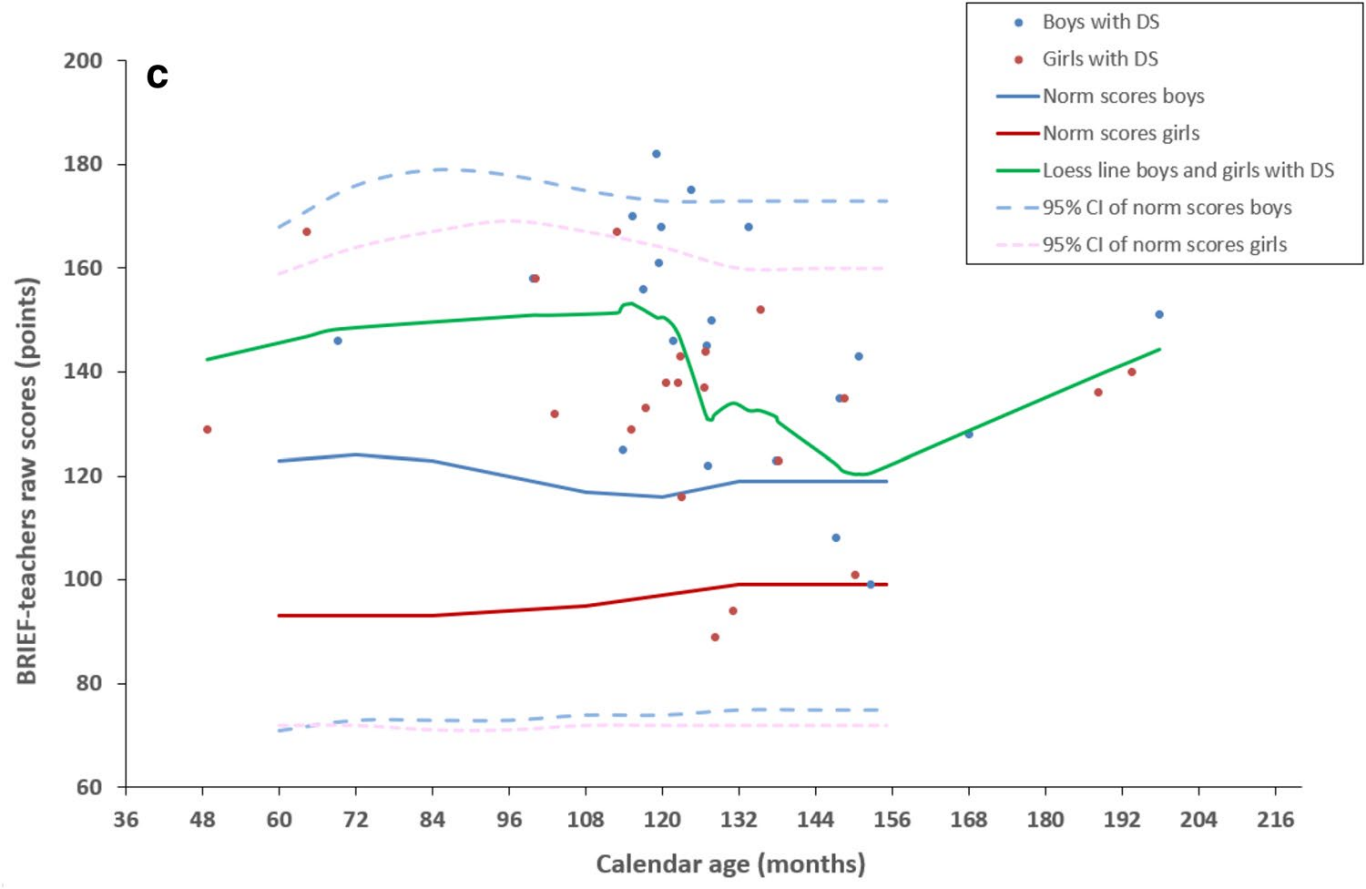
Informant rated executive functions. (**a**) BRIEF-P results for children with DS younger than 8 years (n = 49) Normative data (n = 1747, age 2–5 years) from Gioia et al.^17,18^. **b**) Parent’s version of the BRIEF for children of 8 years and older with DS (n = 89). Normative data (n = 3333, age 5–17 years) from Huizinga et al.^20,21^. **c**) Teacher’s version of the BRIEF for children of 8 years and older with DS (n = 39). Normative data (n = 941, age 5–11 years) from Huizinga et al.^20,21^. Data of children with DS are typically above the norm indicating that children with DS scored poorer on this executive functions scale. Blue bullets: Measured boys with DS (BRIEF-P n = 27, BRIEF parents’ version n = 22, teachers’ version n = 18). Red bullets: Measured girls with DS (BRIEF-P n = 22, BRIEF parents’ version n = 28, teachers’ version n = 21). Green curve: Loess line fitted to the data from ratings on children with DS pooled across boys and girls. Blue curve: Norm scores (mean) of typically developing boys. Red curve: Norm scores (mean) of typically developing girls. Light blue dashed lines: upper and lower bound of the 95% confidence interval of norm scores of typically developing boys. Pink dashed lines: upper and lower bound of the 95% confidence interval of norm scores of typically developing girls.

**Figure 4:**
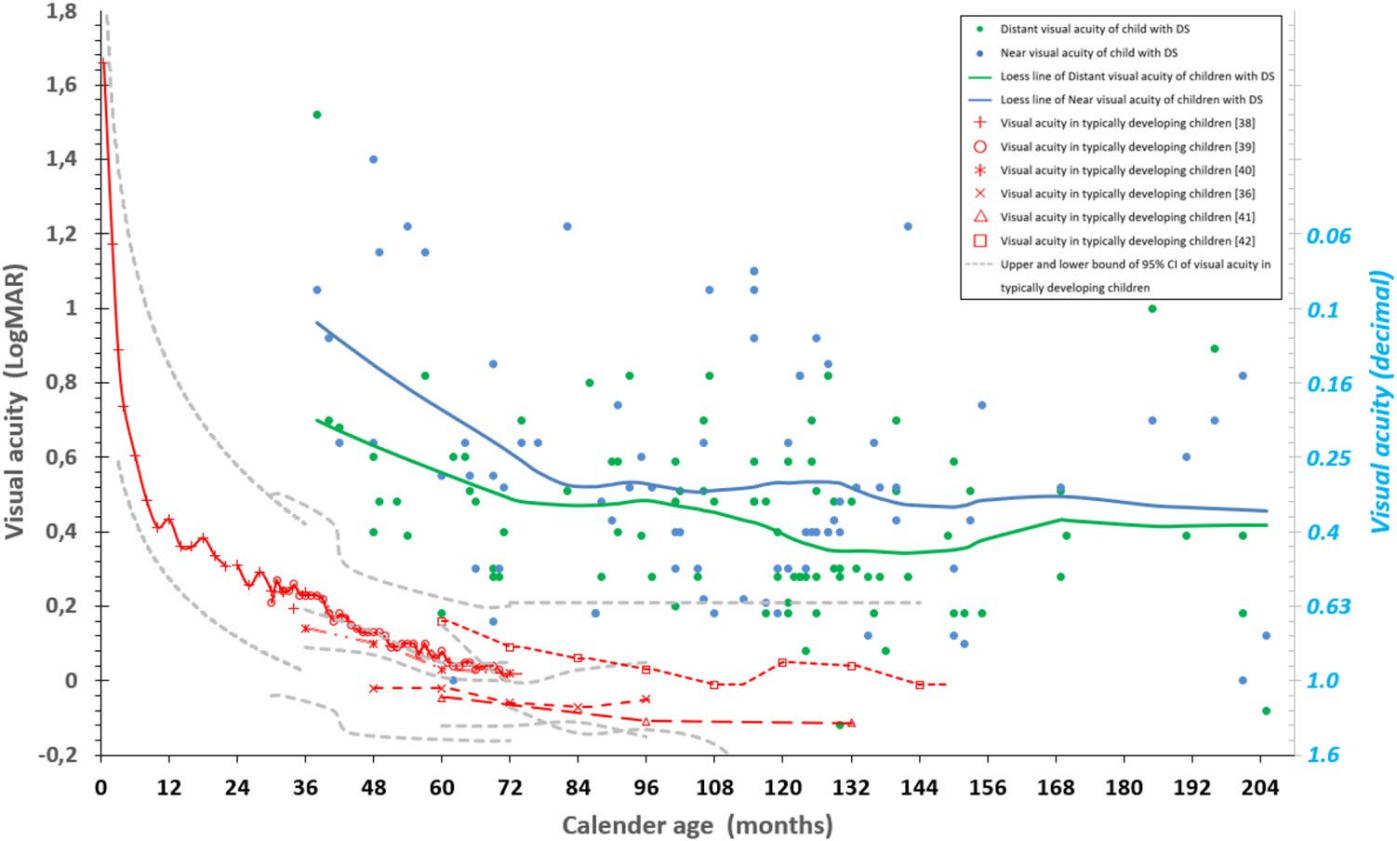
Visual acuity. Normative data from typically developing children (age 0–12 years) are shown in red^70^ with upper and lower bound of the 95% confidence interval in grey dashed lines (extracted from the separate original studies^70^). Note that before the age of 30 months, visual acuity could be estimated only with a preferential looking test such as the Teller acuity chart (TAC)^71^. From the age of 30 months, visual acuity could be assessed with symbols the child named, gestured or matched. Distant (green) and near (blue) visual acuity in our cohort of children with DS as a function of calendar age. Solid lines are Loess lines fitted to the data. Note the gradually improving visual acuity of children with DS and typically developing children. The acuities of the children with DS lie above the norm scores indicating that children with DS scored poorly on visual acuity, near visual acuity being even worse than distant visual acuity (mean difference 0.11 ± 0.32, paired t-test t(73) = -2.900, p = 0.005). Normative distant visual acuities were obtained from: Salomao et al.^38^, (TAC, n = 646, 0–30 months, red crosses), Pan et al.^39^ (HVOT, n = 1722, 30–72 months, red circles), Lai et al.^40^ (Landolt-C, n = 212, 3–6 years, red stars), Huurneman et al.^35^ (Tumbling E, n = 75, 4–8 years, red x-es), Jeon et al.^41^ (Tumbling E, n = 78, 5–11 years, red triangles), Dobson et al.^42^ (ETDRS, n = 252, 5–12 years, red squares). Grey dashed lines: upper and lower bound of the 95% confidence interval of the original norm data.

The scores of the Vineland-S, MEFS as well as the BRIEF-P, BRIEF and visual acuity data were all analysed as a function of calendar age. Pearson correlations or Spearman correlations were computed, and data were compared to norm scores (see Table 1). We used normative data of the Vineland-S^15,16^, n = 979, age range 0–6 years, MEFS^37^, n = 32,800, age range 24 months–18 years, BRIEF-P^17,18^, n = 1747, age range 2–5 years, BRIEF parents’ version^20,21^, n = 3333, age range 5 -17 years and BRIEF teachers’ version^21^, n = 941, age range 5–11 years. The normative data on visual acuity include several studies, total n = 2985, age range 0–12 years: Salomao et al.^38^, n = 646, 0–36 months; Pan et al.^39^, n = 1722, 30 months–6 years; Lai et al.^40^, n = 212, 3–6 years; Huurneman et al.^35^, n = 75, 4–8 years; Jeon et al.^41^, n = 78, 5–11 years; Dobson et al.^42^, n = 252, 5–12 years. For display purposes, a Loess line fitted to the scores of the children with DS was plotted in Figs. 1, 2, 3 and 4.

To compare the data of children with DS to norm scores, we first calculated the difference between the score of the child with DS and the corresponding norm score for that child’s calendar age (i.e., the developmental lag). Thereafter, we analysed the difference scores in a One-sample *t*-test, to test if the mean of the difference scores differed from zero. In a similar way, we also compared the MEFS scores of the children with DS to norm scores corresponding to their Vineland adaptive behavioural age. For this analysis, we first calculated the difference between the score of the child with DS and the norm score matching the child’s adaptive behavioural age. Thereafter, we tested the difference scores in a One-sample *t*-test.

The relation between the developmental lag and calendar age was analysed by linear regression. In the text, B is the estimate of the slope that represents the average change in the dependent variable for a unit change in the independent variable (age).

Visual acuity is typically assessed with uncrowded linearly arranged vision charts at distance. In our cohort, distant visual acuity was assessed with uncrowded linearly arranged optotypes as well. Near visual acuity was assessed in two ways, with uncrowded vision charts and with crowded vision charts. In our analyses, we used the uncrowded distant and near visual acuity assessments (assessed with uncrowded linearly arranged distant and near vision charts). Because uncrowded distant visual acuity equals uncrowded near visual acuity in typically developing children^36^, we used norm scores for distant visual acuity in analyses of near visual acuity as well.

To study the association of developmental lags (difference in scores between children with DS and age-matched norm scores) in adaptive behaviour and executive functions with visual impairments, we used the difference in distant visual acuity (expressed in LogMAR) between children with DS and age-matched norm scores. In multivariate linear regressions of the lags in scores of Vineland adaptive behaviour and the different executive functions assessments, the association with visual impairment was analysed in covariance with age, gender and school attendance. These covariates were chosen according to findings of Papadopoulos et al.^42^ and Metsiou et al.^43^. Information about school attendance was obtained from the parents and was irrespective of duration or type of school. Thereafter, the influence of nystagmus and strabismus was analysed by entering these measures as covariates in the multivariate linear regression.

## Results

Figures 1, 2, and 3 show results from the Vineland-S, the MEFS, the BRIEF-P, and the BRIEF parents’ and teachers’ versions. For completeness, each figure plots the data of boys and girls separately (boys in blue, girls in red), but in our univariate analyses we have pooled the data across gender because there were no significant differences between boys and girls in our cohort.

### Vineland screener questionnaire

The Vineland Screener questionnaires were returned sufficiently filled out by 83 (80%) of the parents.

The Vineland adaptive behaviour (expressed in an age in months) of our participants increased systematically with their calendar age, resulting in a strong positive correlation between the two variables (r = 0.722, p < 0.001; Spearman rank correlation, r = 0.718, p < 0.001) (see Fig. 1). Note, however, that this measure of adaptive behaviour in children with DS fell below the norm score of typically developing children (identity line, derived from n = 979 children)^15,16^. The average difference between Vineland adaptive behaviour and calendar age in children with DS was − 63 ± 35 months (t-test, t(82) = − 16.519, p < 0.001). The magnitude of this developmental lag also correlated with calendar age. With increasing calendar age, the Vineland adaptive behaviour in DS was rated further behind normal (r = 0.965, p < 0.001, B = − 0.78 ± SEM 0.02, R^2^ = 0.931). As inferred from the slope, B, of the regression line, the development is about 80% slower in children with DS compared to the typically developing peers.

To detect possible bias, we compared the mean calendar age in the group of children with a filled in Vineland questionnaire and those without (n = 83 and n = 21, respectively, p = 0.579). We found no differences.

### Minnesota executive function scale

The MEFS was successfully administered in 86 (83%) participants in the age range from 28 to 205 months. In Fig. 2, we first analysed the MEFS data as a function of calendar age and compared the scores of our participants (n = 86) to the normative data of typically developing children (n = 32,800^37^).

The mean MEFS score in the children with DS was 26.2 ± 14.9 points, range 0 to 55, whereas the mean of the corresponding normative scores was 72.3 ± 22.4 points, range 15 to 92. The mean difference between the MEFS scores of children with DS and the norm scores for children of the same calendar age was significant, − 46.1 ± 19.2 points, (*t*-test , t(85) = − 22.246, p < 0.001).

In line with the norm scores, the MEFS scores of participants with DS increased in association with their calendar age (r = 0.484, p < 0.001). The magnitude of the lag in MEFS scores compared to norm scores also correlated with calendar age. With increasing calendar age, the MEFS scores of children with DS lie further below the norm scores (r = 0.684, p < 0.001, B = − 0.32 ± SEM 0.04, R^2^ = 0.47). As inferred from the slope, B, of the regression line, the development of executive functions as assessed with the MEFS is about 30% slower in children with DS.

We also analysed the relation between the MEFS data and adaptive behaviour (expressed as a calendar age in months, estimated by the Vineland-S questionnaire). Towards that end, we compared the MEFS scores of participants with DS (n = 86) to normative data of children of the same adaptive behavioural age. On average, the MEFS scores of the children with DS coincide with the MEFS scores one could expect based on their Vineland adaptive behaviour (mean difference 0.6 ± 13.9 points, *t*-test, t(64) = − 0.339, p = 0.736). Yet, it appeared that the correlation of MEFS scores of DS participants with their Vineland adaptive behaviour (r = 0.545, p < 0.001) was not significantly stronger than the correlation of MEFS scores in DS with their calendar age (r = 0.484, p < 0.001; z = 2.51, p = 0.610).

Children who successfully performed the MEFS test were on average older than those who did not (mean age 109.2 ± 41.5 and 86.6 ± 44.5, t(102) = − 2.075, p = 0.040), and tended to have higher Vineland adaptive behaviour (mean 42.0 ± 12.22 and 35.1, t(81) = − 1.702, p = 0.093).

### BRIEF-P and BRIEF questionnaires

For a total of 89 (86%) children, parents returned the executive functions questionnaires. Some parents completed a questionnaire that did not meet the age range that we had specified, or filled in both BRIEF-P and BRIEF questionnaires. For the analyses below, we used all questionnaire results, provided the number of answered questions exceeded the minima specified in the BRIEF-P and BRIEF protocol (see Table 3b and c).

**Table 3:** Questionnaire return.

Questionnaires executive functions				
(a) Age ranges of questionnaires executive functions				
	Originally designed for age range	Adjusted age range to Down syndrome in our study		
BRIEF-P	2–5 years	2–< 8 years		
BRIEF parents' version	5–18 years	8–18 years		
BRIEF teachers' version	5–12 years	8–18 years		

(b) Number of questionnaires executive functions				
	Questionnaires filled in	Meeting the age range of the study protocol	Not meeting the age range of the study protocol	Missing questionnaires meeting the age range of the study protocol
	n =	n =	n =	n =
BRIEF-P	49	30 (< 8 years)	19 (> 8 years)	11
BRIEF parents' version	50	44 (> 8 years)	6 (< 8 years)	19
BRIEF teachers' version	39	36 (> 8 years)	3 (< 8 years)	27

(c) Number of parents returning combinations of questionnaires				
	Only one questionnaire	Combination with BRIEF-P	Combination with BRIEF parents'	Combination with both BRIEF-P and BRIEF parents'
	n =	n =	n =	n =
BRIEF-P	35			
BRIEF parents' version	11	3		
BRIEF teachers' version	3		25	11

Age ranges and numbers of filled BRIEF-P and BRIEF questionnaires. (a) The age ranges for which the questionnaires were originally designed as well as the adjusted age ranges used of children with DS in the current study. (b) The number of filled in questionnaires. (c) The number of combinations of questionnaires BRIEF-P, BRIEF parents’version and BRIEF teachers’version) which were returned by the parents. n = number of participants.

In Fig. 3, the raw GEC scores of the questionnaires BRIEF-P, BRIEF parents’ and teachers’ versions are plotted as a function of calendar age together with raw GEC norm scores. We found that the scores of children with DS were on average above the norm (poorer executive functions) in all three questionnaires (mean age-matched differences: BRIEF-P, 25.3 ± 17.5 points, *t*-test, t(13) = 5.423, p < 0.001; BRIEF parents’ version, 17.9 ± 26.8 points, *t*-test, t(45) = 4.568, p < 0.001; BRIEF teachers’ version, 40.1 ± 20.5 points, *t*-test, t(28) = 10.535, p < 0.001). None of the scores of our participants correlated with calendar age (all p > 0.197), as was the case with the norm scores. Only the teachers’ ratings on the BRIEF negatively correlated with Vineland adaptive behaviour (r = − 0.368, p = 0.025) indicating that a higher level of adaptive behaviour was associated with better executive functions at school.

In none of the BRIEF-P or BRIEF questionnaires, we found a difference in mean age or mean Vineland adaptive behaviour between the children whose questionnaire was filled in and whose questionnaire was missing.

### Visual acuity

We found poor distant visual acuity in the children in DS as well as a poor near visual acuity. Mean distant visual acuity was 0.43 ± 0.26 LogMAR (~ 0.37 decimal). Mean near visual acuity was 0.56 ± 0.32 LogMAR (~ 0.28 decimal). Their near visual acuity was on average poorer than their distant visual acuity (mean difference 0.11 ± 0.32 LogMAR, paired *t*-test, t(73) = 2.900, p = 0.005). This is in contrast to typically developing children in whom no consistent differences between near and distant visual acuities are found^36^. The difference between near and distant visual acuity in children with DS did not correlate with their calendar age (r = − 0.76, p = 0.520). Visual acuity measurements of our cohort are presented in comparison to the development of (distant) visual acuity during childhood in typically developing children (total n = 2985, age range 0–12 years, including Salomao et al.^38^, n = 646, age range 0–30 months; Pan et al.^39^, n = 1722, age range 30–72 months; Lai et al.^40^, n = 212, age range 3–6 years; Huurneman et al.^35^, n = 75, age range 4–8 years; Jeon et al.^41^, n = 78, age range 5–11 years; Dobson et al.^42^, n = 252, age range 5–12 years) in Fig. 4*.*

The average difference between distant visual acuity in children with DS and distant visual acuity of age-matched typically developing children was 0.51 ± 0.25 LogMAR (~ 0.6 decimal) (*t*-test, t(90) = 19.597, p < 0.001) whereas this difference was 0.63 ± 0.30 LogMAR (~ 0.7 decimal) for near visual acuity (*t*-test, t(75) = 18.175, p < 0.001). These differences did not correlate with calendar age (both p > 0.5).

### Associations between visual and cognitive impairments

We analysed the association of visual impairment (i.e., the difference between visual acuity of children with DS and the age-matched visual acuity norm scores), in particular distant visual impairment, with the lags in cognitive developmental scores of the children with DS (i.e., the difference in scores between children with DS and norm scores of Vineland adaptive behaviour, MEFS scores and BRIEF-P and BRIEF ratings). Following Papadopoulos et al.^44^ and Metsiou et al.^43^, we adjusted for possible confounding factors, age, gender and school attendance. We ran separate multivariate linear regressions on the lags in Vineland adaptive behaviour, MEFS scores and executive function ratings. Reported q-values are p-values adjusted for multiple testing with false discovery rate (FDR) correction.

The lag in Vineland adaptive behaviour was related to visual impairment; milder visual impairment was associated with a smaller lag in Vineland adaptive development (see Table 4).

**Table 4:** Association between visual acuity and Vineland adaptive behaviour.

	r	q	B	SE B
Distant visual impairment	− 0.396	0.001	− 17.37	4.96
Calendar age	− 0.964	< 0.001	− 0.846	0.03
Gender		0.164	0.287	5.48
School attendance	− 0.23	0.055	− 10.69	8.47
Model	− 0.968	< 0.001		

Multivariate analysis of the correlation between the lag in Vineland adaptive behaviour and distant visual acuity impairment adjusting for calendar age, gender and school attendance.

r = partial correlation coefficient.

q = significance of t-test adjusted for multiple testing with false discovery rate (FDR) correction.

B (unstandardized coefficients) = slope.

SE B = standard error of the mean of B.

We analysed the correlation between the developmental lag in Vineland adaptive behaviour and the magnitude of visual impairment, adjusting for their calendar age, gender and school attendance. These impairments were correlated (r = 0.396, q = 0.001). 94% of the variation in the lag of Vineland adaptive behaviour of the child with DS was explained by the model (R^2^ = 0.937, model r = 0.968, q < 0.001). Here, both the magnitude of visual impairment and calendar age had a significant correlation with the lag in Vineland adaptive behaviour (r = − 0.40, q = 0.001, B = − 17.37 ± 5.0 and r = − 0.96, q < 0.001, B = − 0.85 ± 0.029, respectively). Going to school tended to correlate with the lag in Vineland adaptive behaviour (r = − 0.23, p = 0.055, B = − 10.69 ± 8.5). The multivariate linear regression analysis showed that an impairment of one LogMAR line (0.1 LogMAR) in distant visual acuity was associated with a lag of 2 months in Vineland adaptive behaviour. One-month-older calendar age in DS was associated with 0.9 months of additive lag in Vineland adaptive behaviour.

The differences in scores of MEFS, BRIEF-P and BRIEF of the children with DS with respect to the norm scores of typically developing children were not correlated to the magnitude of visual impairment (all p > 0.184).

We also checked for a possible correlation of these cognitive developmental lags with nystagmus and strabismus (the presence of strabismus or size of the manifest angle), two common ocular disorders in DS that can influence visual acuity. In our data, the presence of nystagmus correlated with a lag in distant visual acuity (r = 0.361, p = 0.001). However, in none of these regression analyses on the cognitive developmental lags, we found significant correlations with nystagmus or strabismus.

## Discussion

The current multicentre study investigated adaptive behaviour, executive functions and visual acuity in children with DS, aged 2–16 years, in comparison to the age-matched norm scores of typically developing children. We used a parent-rated questionnaire for adaptive behaviour (Vineland-S) and a combination of parent-rated and teacher-rated questionnaires (BRIEF-P and BRIEF) as well as a task-based test (MEFS) for assessing executive functions. Visual acuity was assessed with symbol discrimination on visual acuity charts at distance and at near.

Compared to typically developing children, children with DS had a lower outcome on adaptive behaviour (Vineland-S), poorer outcome on executive functions according to both task-based (MEFS) and rating-based (BRIEF-P and BRIEF) assessment, and poorer visual acuity. Their near visual acuity was even worse than their distant visual acuity. Moreover, the lag in Vineland adaptive behaviour of children with DS was related to the severity of their visual impairment.

### Adaptive behaviour

In line with previous studies^34,45–50^, the children with DS in our study had weaker adaptive behaviour than typically developing children. As found by Papadopoulos et al.^44^ in children with isolated visual impairments (i.e., without DS), older children with DS performed better in the current study too. But, they also showed a larger difference with typically developing children in comparison to younger children with DS. We found the lowest scores in adaptive behaviour in children with poorest visual acuities. This new finding in children with DS is in line with the reports of children with isolated visual impairment (without DS) by Sonckson and Dale^7^, Dale and Sonckson^8^, Tadic et al.^13^ and Bathelt et al.^11^.

Even when we analysed the influence of visual acuity in covariance with age, we found the lowest performance in the children with DS with the poorest visual acuities. This agrees with findings of Bathelt et al.^11^. They found poorer adaptive behaviour—in practical, social and conceptual composite scores—in children (without DS) with ascending levels of isolated visual impairment including mild to moderate and severe to profound visual impairments.

As in afore mentioned studies in children with isolated visual impairment^7,8,11,13^, we only used the assessments of distant visual acuity. Separate analyses of the influence of near visual acuity were omitted because there were too many missing data.

### MEFS and BRIEF-P and BRIEF executive functions

In our study, children with DS had poorer executive function-outcomes than typically developing children in both task-based scores and in informant report ratings. This agrees with previous studies^51–57^. In executive functioning assessed with the MEFS, we found an association with calendar age. Older children with DS obtained higher MEFS scores, but they showed larger differences with norm data than younger children with DS. The scores show a large difference with age-matched norm scores, but this reduced MEFS performance is in line with the performance one can expect from their adaptive behaviour (estimated with Vineland-S questionnaire and expressed in age, months). The lack of correlation of BRIEF-P and BRIEF ratings with calendar age in our study agrees with findings of Lee et al.^57^ and the lack of correlation with age in norm data with findings of Gioia et al.^17^ and (Huizinga et al.^21,22^).

The MEFS scores of the children with DS were not clearly associated with their visual acuity. Thus, the MEFS is suitable for children with visual impairment even though some vision is required to do the test.

### Relation between adaptive behaviour scores and executive functions scores

Children with poorer adaptive behaviour showed a lower score on the MEFS, which is in line with a recently published study in children with DS by Sabat et al.^58^. The assessments they used resemble the combination of a questionnaire and a card sorting test, MEFS, we used: ABAS-II parents’ and teachers’ version^59^, and three executive functions tasks including the Wisconsin Card Sorting Test^60^, respectively. Other researchers^56^ also reported weaker performance on task-based executive functions tests in children with DS compared to children with normal development. In our study, we found an inverse correlation of Vineland adaptive behaviour with BRIEF-teachers’ version but not with the parents’ versions of the BRIEF and BRIEF-P questionnaires. Explanations for these discrepancies between ratings of executive functions by teachers and by parents include the possibility that teachers and parents are observing different behaviours or phenotypes. The more structured demands of school settings versus relatively less organized home activities may challenge the children in a different way^61,62^. So, different informants may validly contribute unique information from different perspectives.

In the current study, the MEFS scores of children with DS were comparable to the norm scores of typically developing children with the same level of adaptive behaviour. This relation shows the robustness of the Vineland-S and MEFS and underlines the suitability of the MEFS for use in children with DS.

### Visual acuity, distant and near

The differences in distant visual acuity and in near visual acuity between children with DS and norm scores—poorer scores in children with DS—agree with previous publications^5,63^. These differences did not change with age because of a similar improvement with age in children with DS and in norm scores. Visual acuity develops slowly (see Fig. 4). Among other factors, the quality of the image on the retina in childhood ages is very important^64,65^. The image quality on the retina can be optimized by correction of refractive errors. In the current study, the children wore their habitual glasses or no corrections at all (i.e., usual care). But, this may not have been the optimal situation because the refractive error can change more rapidly in children with DS than in typically developing children^66^. However, children with DS do not complain because of low visual acuity. So, the need for adjustment of glasses may go unnoticed, unless regular screening is performed. In the literature, this was analysed by van Splunder et al.^67^. Prior to their large study on visual impairment in 1598 adults with DS in the Netherlands, visual impairment or blindness had remained undiagnosed in 40.6% of the persons. By contrast, typically developing children frequently do complain when their corrections are not optimal anymore. We, therefore, can expect that the typically developing children, from whom the norm scores were derived, getting usual care, had better corrections compared to the children with DS. Thus, non-optimal correction of refractive errors by suboptimal glasses may have played a role in the large difference in visual acuity between children with DS and typically developing children.

Apart from non-optimal corrections, other factors may have played a role. The abnormal morphology of the visual cortex in children with DS^4^ induces impairment in cerebral visual processing, so called cerebral visual impairment (CVI)^68^. Thus, probably, the differences in visual acuity between children with DS and typically developing children also are the result of CVI in children with DS. CVI includes accommodation lags and crowding problems^69^. Accordingly, we found accommodation lags resulting in low near visual acuity and the poor crowded near visual acuity in all of our participants (see Table 2).

The observed difference between distant and near visual acuity in children with DS is mainly due to accommodation lags with non-optimal refractive corrections. In our RCT, we found that the difference between distant and near visual acuity in children with DS can be minimized with optical corrections tailored to the ocular disorders of children with DS^27^. Wearing full correction of refractive error can maximize distant visual acuity and partly support near visual acuity. Additionally, wearing bifocal glasses, the extra correction for looking at short distances stimulates the development of near visual acuity^27^ and thus reduces the difference between distant and near visual acuity in children with DS.

### Strengths and limitations

Strengths of the current study include: the large sample size with a relatively rare and biologically well-defined condition (DS), the robust and standardised measurements that made data collection across multiple sites possible, the use of both task-based and informant-based measures of executive function, and the consideration of both adaptive developmental age (estimated from their adaptive behaviour in the Vineland-S questionnaire) and calendar age. In addition, this is the first study in children with DS to assess task-based executive functions with the MEFS.

Limitations of the study include the fact that normative data, being derived from other studies, may have been collected under different experimental conditions. However, all these studies included large groups (see Table 1). Norm scores of the Vineland-S and BRIEF-P had a limited age range (from 0 to 6 years and from 2 to 5 years, respectively), which limited comparisons with older children. Furthermore, as 90% of the included children needed updated glasses^27^, a variable part of the visual impairments may have been due to insufficient correction of refractive errors. These avoidable impairments might have obscured a relation between executive functioning and the level of best-corrected visual acuity.

We also encountered difficulties in the acquisition of data, resulting in missing data. This was partly due to practical issues (no iPad with the MEFS test available), but mostly due to a lack of cooperation of the participants during the assessments on the one hand, and parental inattentiveness in returning completed questionnaires on the other. It is known that in children with DS, as in other children with cognitive disabilities, cooperation difficulties can emerge. In our cohort of children with DS, in which children were not selected because of high level functioning or cooperation, these difficulties were unavoidable. The local examiners all had volunteered to cooperate in this study and did their utmost to collect the data. Despite their motivation to obtain the necessary measurements, they sometimes had to skip a test, or stop the measurements according to the Dutch code of conduct relating to expressions of objection by people who are incapable of giving consent (2002). However, of all the scores we collected, only the MEFS scores may have been affected by an age difference between the group of children whose scores were available and whose data were missing. By including young children with DS regardless of their developmental delay, it was unavoidable that we encountered children whose adaptive behaviour was not proficient enough to perform the MEFS test (designed for children without cognitive delay from the age of 2 years).

Finally, a cross-sectional analysis, as was performed in the current paper, has inherent limitations. It compares participants to estimate the development of skills with increase of age. In this particular case, it is obvious that the sample of children with DS is much more heterogeneous in terms of achieving adaptive skills or executive functions than a non-clinical typical developing sample. This clearly underlines the need for a longitudinal design in order to understand better the development of adaptive behaviour and executive functions in children with DS and its relation with visual acuity.

### Implications of the differences found between DS and typically developing children

Children with DS attend regular schools or special schools, both with only a minority of children with DS. Being aware of the specific limitations in abilities of this syndrome can be a support to parents, teachers and other professionals in the management and guidance of children with DS. Regular, targeted screening can detect, specify and quantify the developmental lags and estimate the specific need of the individual child with DS. Interventions or adaptations might be developed and applied to support and stimulate development to, at least partly, reduce the developmental lags in children with DS. Optimizing visual functions with corrective glasses tailored to the ocular disorders of children with DS is one of these interventions. Possibly, besides improving visual functions, it may stimulate development on different levels, including adaptive behaviour and executive functions. A cumulative impact of one of the delays in development on other developmental processes, already mentioned and shown in children with isolated visual impairments^7,8,13^, might also exist in children with DS. In the current study, a significant relation between visual acuity impairment and a lag in Vineland adaptive behavioural was found. It has still to be proven that interventions are useful in decreasing differences, which otherwise, according to our findings, get larger with increasing age. It is also not yet clear what the developmental range is in children with DS with optimal corrections and interventions.

### Conclusions

Children with DS in the age range of 2–16 years are severely impaired in adaptive behaviour, executive functions and visual acuities (both distant and near). Larger impairments in Vineland adaptive behaviour are associated with larger impairments in visual acuity. This supports the idea that visual acuity plays a role in the development of adaptive behaviour, as previously suggested for visually impaired children without known developmental disorders. Furthermore, near visual acuity is more severely impaired in DS than distant visual acuity, presumably because of the accommodation lag in DS. These findings emphasize the necessity of regular screening during development in DS and, if possible, the application of interventions or adaptions.
